# Diagnostic and grading accuracy of ^18^F-FDOPA PET and PET/CT in patients with gliomas: a systematic review and meta-analysis

**DOI:** 10.1186/s12885-019-5938-0

**Published:** 2019-08-05

**Authors:** Jiarui Xiao, Yizi Jin, Ji Nie, Fukun Chen, Xuelei Ma

**Affiliations:** 1 Department of Biotherapy, Cancer Center, State Key Laboratory of Biotherapy, West China Hospital, Sichuan University and Collaborative Innovation Center, No.37, Guoxue Alley, Chengdu, 610041 Sichuan China; 2 Medical College, Soochow University, Suzhou, 215006 Jiangsu China; 3 Department of Nuclear Medicine, Yunnan Cancer Hospital, the Third Affiliated Hospital of Kunming Medical University, Kunming, 650118 Yunnan China; 4 West China School of Medicine, Sichuan University, Chengdu, 610041 Sichuan China

**Keywords:** PET, ^18^F-FDOPA, Glioma, Meta-analysis

## Abstract

**Background:**

Positron emission tomography (PET) and PET/computed tomography (PET/CT) imaging with 3,4-dihydroxy-6-[^18^F] fluoro-L-phenylalanine (^18^F-FDOPA) has been used in the evaluation of gliomas. We performed a meta-analysis to obtain the diagnostic and grading accuracy of ^18^F-FDOPA PET and PET/CT in patients with gliomas.

**Methods:**

PubMed, Embase, Cochrane Library and Web of Science were searched through 13 May 2019. We included studies reporting the diagnostic performance of ^18^F-FDOPA PET or PET/CT in glioma patients. Pooled sensitivity, specificity, and area under the summary receiver operating characteristic (SROC) curve were calculated from eligible studies on a per-lesion basis.

**Results:**

Eventually, 19 studies were included. Across 13 studies (370 patients) for glioma diagnosis, the pooled sensitivity and specificity of ^18^F-FDOPA PET and PET/CT were 0.90 (95%CI: 0.86–0.93) and 0.75 (95%CI: 0.65–0.83). Across 7 studies (219 patients) for glioma grading, ^18^F-FDOPA PET and PET/CT showed a pooled sensitivity of 0.88 (95%CI: 0.81–0.93) and a pooled specificity of 0.73 (95%CI: 0.64–0.81).

**Conclusions:**

^18^F-FDOPA PET and PET/CT demonstrated good performance for diagnosing gliomas and differentiating high-grade gliomas (HGGs) from low-grade gliomas (LGGs). Further studies implementing standardized PET protocols and investigating the grading parameters are needed.

## Background

Glioma is the most common primary brain tumor, accounting for 81% of all malignant brain tumors with an annual incidence of 5.26 per 100,000 individuals [1]. According to the World Health Organization (WHO) 2007 classification, grade I and II tumors are together referred to as low-grade gliomas (LGGs), while grade III and IV tumors are categorized into high-grade gliomas (HGGs) [2]. The treatment of gliomas requires multidisciplinary care. Appropriate surgical or radiotherapy regimen is highly dependent on the delineation and grade of tumors, and therefore imaging assessment is critical to the clinical management of affected patients. For the past few decades, conventional magnetic resonance imaging (MRI) has been the method of choice for glioma diagnosis. However, it lacks sensitivity in non-enhancing gliomas and cannot reliably provide the differentiation between tumor recurrence and radiation-induced changes (e.g., pseudoprogression and radionecrosis) [3, 4]. Thus, more accurate imaging modalities need to be found.

The Response Assessment in Neuro-Oncology (RANO) working group has recently recommended the use of positron emission tomography (PET) imaging for gliomas complementary to MRI [5]. Compared with MRI, PET provides additional insight into tumor metabolism and has been shown to improve tumor delineation and grading [6]. The glucose metabolic agent 18-fluoro-deoxyglucose (^18^F-FDG) has been the classic PET tracer used in tumor imaging, but it is not ideal in detecting gliomas due to the high physiologic uptake in normal brain tissue. Given the diagnostic limitations of ^18^F-FDG, amino acid tracers have been extensively investigated, such as 3,4-dihydroxy-6-[^18^F] fluoro-L-phenylalanine (^18^F-FDOPA). Unlike gadolinium, ^18^F-FDOPA is transported across the intact blood brain barrier (BBB) [7]. Hence the use of ^18^F-FDOPA PET enables the depiction of tumor components beyond contrast enhancement in MRI [8]. Furthermore, the accuracy of ^18^F-FDOPA PET and PET/CT in detecting gliomas has been reported to be superior as compared with ^18^F-FDG PET and PET/CT in previous studies [9, 10]. However, existing studies are inconclusive because of relatively small sample sizes and heterogeneous designs. Also, previous studies have provided contradictory conclusions on whether there are significant differences in ^18^F-FDOPA PET and PET/CT between low-grade gliomas (LGGs) and high-grade gliomas (HGGs) [11–13].

We performed this meta-analysis to systemically review all relevant publications in attempt to (1) evaluate the overall diagnostic performance of ^18^F-FDOPA PET and PET/CT in patients with gliomas and to (2) access the ability of ^18^F-FDOPA PET and PET/CT in discriminating HGGs from LGGs.

## Methods

### Search strategy

A systematic search of the PubMed, Embase, Cochrane Library and Web of Science databases was performed for English and non-English publications through 13 May 2019 using the following search: “(DOPA [all fields] OR FDOPA [all fields] OR fluorodihydroxyphenylalanine [all fields]) AND (positron emission tomography [all fields] OR PET [all fields]) AND (glioma [all fields] OR gliomas [all fields] OR brain tumor [all fields] OR brain tumors [all fields])”. References to retrieved articles and unpublished clinical trials were also checked for potential findings.

### Study selection

All records were screened independently for eligibility by 2 reviewers (JX and YJ), and discrepancies were resolved by consensus. The inclusion criteria were: (1) Original studies investigating the diagnostic or grading capacity of ^18^F-FDOPA PET or PET/CT in patients with gliomas; (2) Studies with histopathology and/or clinical and imaging follow-up as reference standards; (3) Certain numbers of true-positive (TP), false-positive (FP), false-negative (FN) and true-negative (TN) results in diagnostic or grading tests can be derived from sufficient data. Case reports, reviews, letters and in vitro studies were excluded. Meanwhile, studies involving diagnosing brain metastases were also excluded (unless single cases can be differentiated). When study populations overlapped, we only included the most recent one to avoid data duplication [14, 15]. Eligible literatures were then classified into two different groups according to different aims of study: (1) Studies focused on the diagnosis of gliomas; (2) Studies focused on the grading of gliomas.

### Data extraction and quality assessment

Two reviewers (JX and YJ) independently went through all eligible studies and extracted essential information, including name of principal author, year of publication, study country, type of study design (retrospective or prospective), number of specimens, number of patients, mean or median age of patients, male to female ratio, index test, prior treatment, tumor occurrence (newly diagnosed or recurrent), reference standard, comparative imaging approaches, parameters and threshold (if existed), and the number of TP, FP, FN, TN.

Quality Assessment of Diagnostic Accuracy Studies 2 (QUADAS-2) [16] was applied to evaluate the quality of all included studies in Review Manager 5.3 software (Cochrane Collaboration, Oxford, England). QUADAS-2 tool is composed of four aspects, of which each item can be defined as “yes”, “no” or “unclear”. The overall assessments of each aspect interpret the bias risk as low, high or unclear. Two reviewers (JX and YJ) independently assessed each article with disagreements resolved.

### Statistical analysis

Meta-DiSc software, version 1.4 (Clinical Biostatistics Unit, Ramón y Cajal Hospital, Madrid, Spain) was used to calculate pooled data including sensitivity, specificity, positive likelihood (LR+), negative likelihood (LR-), diagnostic odds ratio (DOR) (with 95% confidence intervals (CI)), and construct summary receiver operating characteristic (SROC) curves [17]. The Area Under the Curve (AUC) was computed to measure overall performance of tests (AUC is positively correlated with diagnostic value, 0.5 ≤ AUC < 0.7 representing a low accuracy; 0.7 ≤ AUC < 0.9 indicating a moderate accuracy; AUC ≥ 0.9 indicating a high diagnostic value) [18, 19]. StataSE, version 12 (StataCorp, College Station, TX, USA) was employed to assess publication bias through Deeks’ Funnel Plot Asymmetry Test [20]. The Spearman’s rank correlation coefficient was calculated between logarithmic sensitivity and logarithmic (1-specificity), and a strong positive correlation indicates the existence of threshold effect [17]. We used Chi-square, Cochran-Q, and I-squared test to evaluate the heterogeneity between studies [17]. The Random-Effects Model would be applied, unless no significant heterogeneity was detected between studies [21].

Considering the differences between primary and recurrent tumors in clinical management and post-treatment changes, we divided all studies into two subgroups: detecting (1) newly diagnosed gliomas and (2) recurrent (including residual) gliomas. TP, FP, FN, TN were recalculated in studies investigating in a mixed population of primary and recurrent gliomas when feasible. (Significant level: two-tailed *p*-value< 0.05).

## Results

### Study selection

**Fig. 1:**
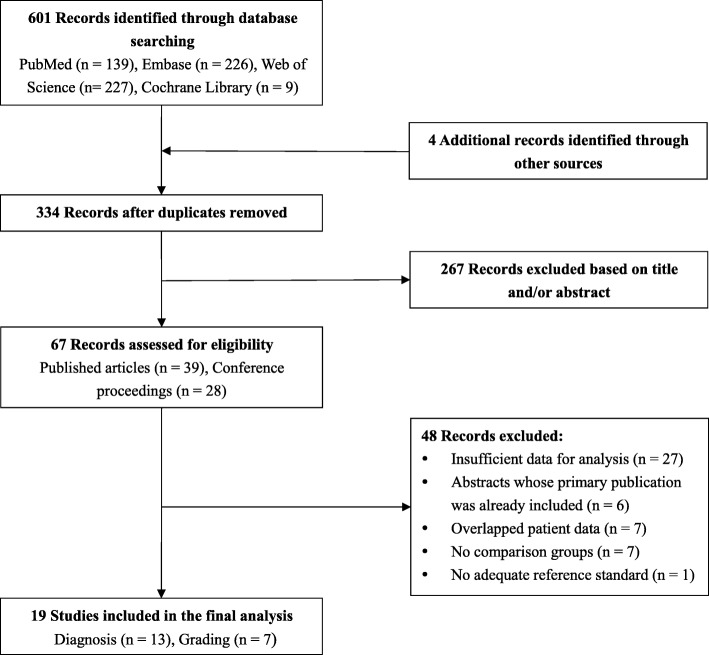
Flow chart of study selection. 19 studies are included eventually

The comprehensive literature search yielded 605 relevant records in total. After excluding duplicates and screening through titles and abstracts, 67 records went into full-text review, with 19 studies fulfilling the inclusion criteria for the final meta-analysis. Eligible studies were further classified for the intended subanalyses: 13 studies for glioma diagnosis and 7 studies for glioma grading. The details of the selection process are illustrated in Fig. 1.

### Study characteristics

The characteristics of the included studies are demonstrated in Table 1 and Table 2.

**Table 1:** Baseline information of included studies for glioma diagnosis

Reference	Year	Country	Design	Specimens No.	Patients No.	Age, yr		M/F	Test	Prior treatment	Occurrence	Gold Standard
						Mean^a^	Median					
Chen et al. [9]	2006	US	Prospective	27	30 (27^b^)	–	–	–	PET	With or without	7 New+ 20 Recur	Histo+Radio+follow-up
Tripathi et al. [10]	2009	India	Prospective	15	15	28.4 ± 11.1	–	9/6	PET/CT	With (Sx/CT/RT) or without	3 New+ 12 Recur	Histo+Radio+follow-up
Sellam et al. [22]	2010	India	Prospective	30	30	–	–	–	PET/CT	Sx+/−RT	Recur	Histo+Radio+follow-up
Jora et al. [23]	2011	India	Prospective	23	23	43.25 ± 14.9	–	–	PET/CT	15 with (Sx + RT) + 8 without	8 New+ 15 Recur	Histo+Radio+follow-up
Karunanithi et al. [15]	2013	India	Prospective	35	35	36.62 ± 0.86	–	28/7	PET/CT	Sx + RT+/−CT	Recur	Histo+Radio+follow-up
Pafundi et al. [24]	2013	US	Prospective	23	10	40.8 ± 18.9	–	9/1	PET/CT	With or without	8 New+ 2 Recur	Histo
Herrmann et al. [25]	2014	US	Retrospective	110	110	51.7 ± 12.1	52.5	72/38	PET/CT	Sx	Recur	Histo+Radio+follow-up
Moran et al. [26]	2015	Italy	Retrospective	27	27	10	10	15/12	PET	1 with (Sx + CT + RT) + 20 without	20 New+ 1 Recur	Histo+Radio+follow-up
Sharma et al. [27]	2016	India	Prospective	12	11	34	–	6/5	PET/CT	Sx	Recur	Histo+Radio+follow-up
Paquet et al. [28]	2017	France	Prospective	60	35	60	–	–	PET	Sx + CT + RT	Recur	Histo+Radio
Evangelista et al. [29]	2018	Italy	Retrospective	13	13	–	60	–	PET/CT	Unclear	Recur	Radio+follow-up
Youland et al. [30]	2018	US	Prospective	37	13	–	40	9/4	PET	Sx/CT/RT	Recur	Histo
Evangelista et al. [31]	2019	Italy	Retrospective	21	21	58 ± 11	–	–	PET/CT	Sx + CT/RT/immunotherapy	Recur	Radio+follow-up

*M* male, *F* female, *New* newly-diagnosed, *Recur* recurrent, *Histo* histopathology, *Radio* radiology, *Sx* surgery, *CT* chemotherapy, *RT* radiation therapy

^a^Mean age is expressed as mean ± standard deviation

^b^Three patients with brain metastases were excluded

**Table 2:** Baseline information of included studies for glioma grading

Reference	Year	Country	Design	Specimens No.	Patients No.	Age, yr		M/F	Test	Prior treatment	Occurrence	Gold Standard	Parameter	Cut-off
						Mean^a^	Median							
Fueger et al. [12]	2010	US	17 Prospective+ 42 Retrospective	59	59 (22^b^)	–	44.5	13/9	PET or PET/CT	With (Sx + CT/RT) or without	New	Histo	SUVmax	2.72
Nioche et al. [32]	2013	France	Prospective	33	33	51 ± 16	51	28/5	PET/CT	With (Sx + CT/RT) or without	20 New+ 13 Recur	Histo	SUVmean	2.2
Pafundi et al. [24]	2013	US	Prospective	23	10 (9^b^)	42.9 ± 19.2	–	8/1	PET/CT	With or without	7 New+ 2 Recur	Histo	SUVmax T/SUVmean N	2.0
Janvier et al. [13]	2015	France	Retrospective	31	31	36.8 ± 12.1	–	13/18	PET	With (Sx/CT/RT) or without	25 New+ 6 Recur	Histo+Radio+follow-up	SUVmean T/N	1.33
Bund et al. [33]	2017	France	Prospective	53	53	38	–	23/30	PET/CT	Without	New	Histo	SUVmax T/N	2.16
Morana et al. [34]	2017	Italy	Retrospective	26	26	10.2 ± 4.6	9.5	15/11	PET	Without	New	Histo	SUVmax T/S	0.90
Patel et al. [35]	2018	US	Prospective	45	45	46.4 ± 16.2	–	22/23	PET	Without	New	Histo	SUVmax T/N	1.7

M, male; F, female; Sx, surgery; CT, chemotherapy; RT, radiation therapy; New, newly-diagnosed; Recur, recurrent; Histo, histopathology; SUV, standardized uptake value; Radio, radiology; T, tumor; N, normal; S, Striatum

^a^Mean age is expressed as mean ± standard deviation

^b^Patients that are finally included in the quantitative analysis by authors

Aim 1: Investigating the accuracy of ^18^F-FDOPA PET and PET/CT for diagnosing gliomas.

Altogether, 13 studies (370 patients) [9, 10, 15, 22–31] were included in this meta-analysis. 4 of the 13 studies (31%) were retrospective studies, whereas the others (69%) were designed prospectively. Other than Morana et al. [26], which focused on the diagnostic performance of ^18^F-FDOPA PET on pediatric gliomas, all researches were carried out among adults. 8 of 13 studies focused only on diagnosing recurrent gliomas with previous treatment, while the rest studies were conducted jointly in newly diagnosed and pretreated patients, and all of them can be further stratified into corresponding subgroups. All TP, FP, FN and TN results extracted were based on visual analysis.

**Fig. 2:**
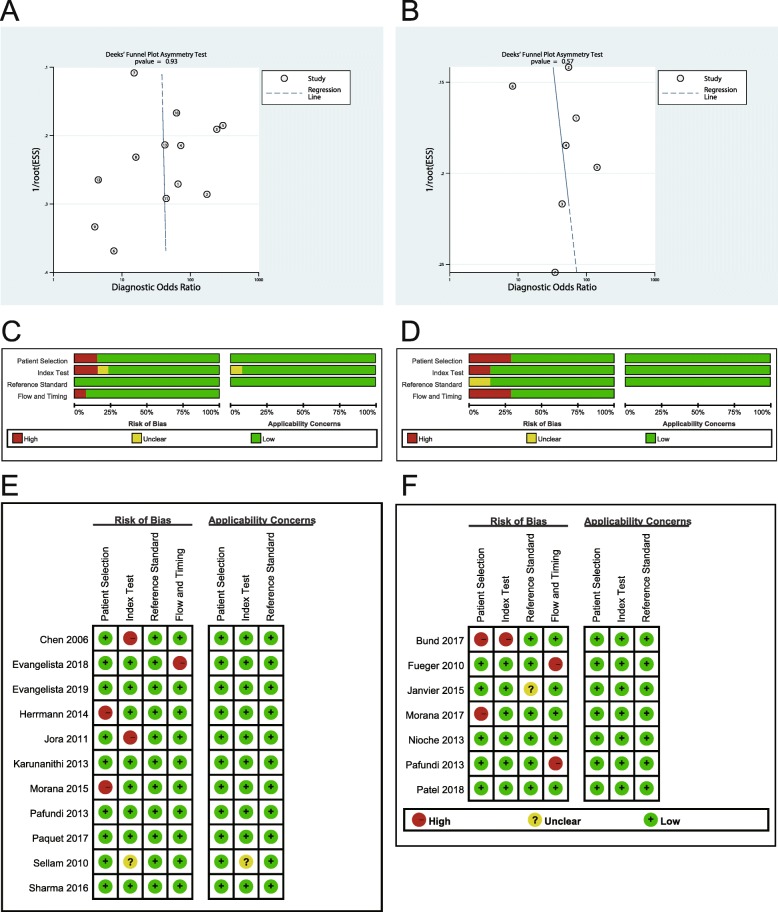
Publication bias assessment of included studies (**a**, **b**) and quality assessment of included studies (**c**-**f**). **a**, **b**: Deeks’ Funnel Plot shows no publication bias in both detecting (**a**) and grading (**b**) gliomas. **c**-**f**: The graphs show risk of bias and applicability concerns regarding each study. Quality assessment result for diagnosis (**c**, **e**); for grading (**d**, **f**)

Spearman correlation coefficient was − 0.18 (*p*-value = 0.56), displaying no threshold effect. Deeks’ Funnel Plot test demonstrated no publication bias (*p*-value = 0.93, Fig. 2a).

Aim 2: Investigating the performance of ^18^F-FDOPA PET and PET/CT for grading gliomas.

As for the meta-analysis of grading gliomas, 7 studies [12, 13, 24, 32–35] with 219 patients were involved in total, with male/female ratio of 122/97 (1.26). 193 (88%) patients were newly diagnosed, and 26 (12%) patients were diagnosed with recurrent gliomas. All studies were performed at the patient level. The number of TN represents the number of discriminating HGGs from LGGs. All the interpretations were based on quantitative analysis, and the cut-off value of each study was determined through ROC analysis. For those used various parameters, the most predictive index was included in this analysis.

Neither threshold effect nor publication bias was detected through Spearman correlation coefficient (= 0.67, *p*-value = 0.10) and Deeks’ Funnel Plot test (*p*-value = 0.57, Fig. 2b) respectively.

### Quality assessment

We used QUADAS-2 to estimate the quality of literature in Review Manager 5.3. The assessment results were shown in Fig. 2(c-f).

Meta-analysis of Diagnostic/Grading Performance in Gliomas.

Aim 1: Investigating the accuracy of ^18^F-FDOPA PET for diagnosing gliomas.

**Fig. 3:**
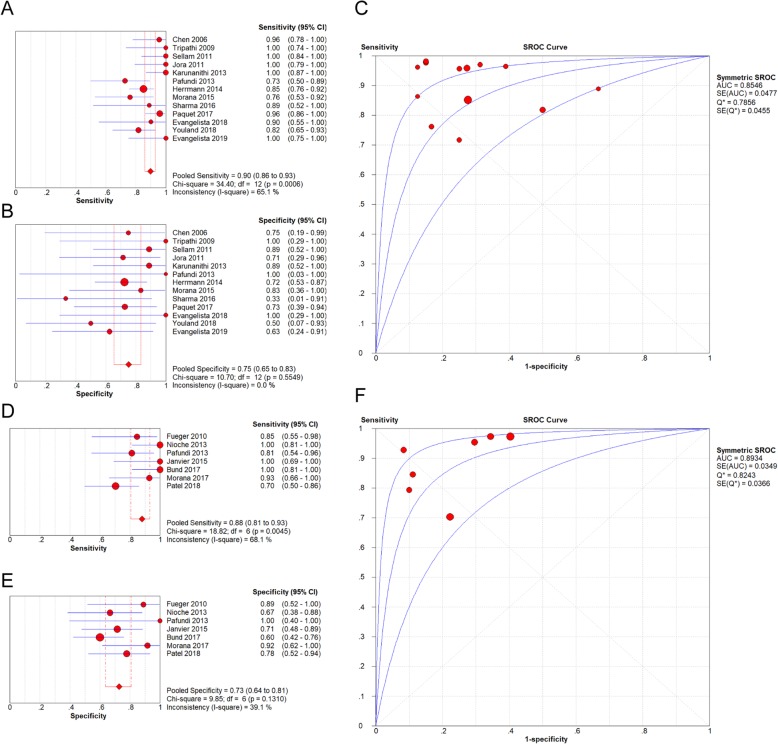
Forest plots of sensitivity and specificity, and summary receiver operating characteristic curve of diagnosing gliomas (**a**-**c**) and differentiating HGGs from LGGs (**d**-**f**)

Meta-analysis resulted in a pooled sensitivity of 0.90 (95% CI: 0.86–0.93), and a pooled specificity of 0.75 (95% CI: 0.65–0.83). The pooled LR+ was 2.84 (95% CI, 2.09–3.85), and the pooled LR- was 0.15 (95% CI: 0.09–0.26). The DOR was 24.05 (95% CI: 12.62–45.85) (Fig. 3a/b).

SROC curve was established based on pooled sensitivity and specificity, and the overall AUC was 0.85 [Standard Error (SE) = 0.05], indicating a moderately high overall diagnostic value of ^18^F-FDOPA PET and PET/CT on gliomas among individuals (Fig. 3c).

Only a significant heterogeneity for sensitivity was detected (Chi-square = 34.40, *p*-value = 0.0006, I-square = 65.1%) through Chi-square, Cochran-Q, and I-squared tests (Fig. 3a).

### Subgroup analysis

No threshold effect was detected in two subgroups. Pooled data and SROC curve manifested a high diagnostic value of ^18^F-FDOPA PET and PET/CT in recurrent subgroup, with sensitivity of 0.92 (95% CI: 0.88–0.95), specificity of 0.76 (95% CI: 0.66–0.85), LR+ of 2.87 (95% CI: 2.01–4.11), LR- of 0.13 (95% CI: 0.07–0.23), DOR of 29.65 (95% CI: 13.09–67.15), and AUC of 0.90 (SE = 0.06). In the newly-diagnosed subgroup, only two studies were eligible for meta-analysis (in another three studies only sensitivity can be computed after stratification). In comparison with recurrent group, pooled data of newly-diagnosed group revealed a lower sensitivity of 0.71 (95% CI: 0.54–0.85) and a higher specificity of 0.86 (95% CI: 0.42–1.00). The LR+, LR- and DOR were 3.71 (0.87–15.81), 0.36 (0.19–0.68) and 10.88 (1.57–75.31) respectively.

Aim 2: Investigating the Performance of ^18^F-FDOPA PET and PET/CT for Grading Gliomas.

As illustrated in the Fig. 3(d-f), ^18^F-FDOPA PET and PET/CT presented a pooled sensitivity of 0.88 (95% CI: 0.81–0.93), specificity of 0.73 (95% CI: 0.64–0.81), LR+ of 2.90 (95% CI: 2.19–3.85), LR- of 0.16 (95% CI: 0.08–0.36), DOR of 25.87 (95% CI: 10.53–63.54), and AUC of 0.89 (SE = 0.03) for differentiating HGGs from LGGs.

Other than sensitivity (*p*-value = 0.0045), no significant heterogeneity was found among studies for specificity (*p*-value = 0.13), LR+ (*p*-value = 0.42), LR- (*p*-value = 0.07) and DOR (*p*-value = 0.53).

## Discussion

This meta-analysis demonstrated a pooled sensitivity of 0.90 and specificity of 0.75 for ^18^F-FDOPA PET and PET/CT in diagnosing gliomas, and a pooled sensitivity of 0.88 and specificity of 0.73 in grading gliomas. Subgroup analysis showed that ^18^F-FDOPA PET and PET/CT had good diagnostic performance in both newly-diagnosed and recurrent groups. The specificity for detecting recurrent gliomas was slightly lower, which may be explained by the increased false positive rate caused by treatment response such as edema and inflammatory tissue. However, it is worth mentioning that the limited number of studies in newly-diagnosed group may impair the reliability to some degree.

The diagnostic accuracy of ^18^F-FDOPA PET for distinguishing radiation necrosis from brain tumor recurrence has been previously investigated by Yu J et al. [36]. However, the use of ^18^F-FDOPA PET or PET/CT in accessing newly-diagnosed gliomas has not been systematically studied before. Moreover, the recommendations for the use of ^18^F-FDOPA in glioma grading remained controversial [5]. Our findings confirmed there is a meaningful role of 18F-FDOPA PET and PET/CT for the evaluation of glioma patients.

Of increased interest is the value of ^18^F-FDOPA PET and PET/CT in differentiating tumor recurrence from radiation-induced changes. At present, MRI remains the standard imaging method for assessing treatment effects in glioma patients [37]. However, the utility of contrast enhancement MRI mainly relies on the disruption of blood-brain barrier (BBB), which impairs its specificity in differentiating radiation-induced changes from recurrent or residual brain tumors, such as pseudoprogression and radionecrosis. New amino acid tracers including ^18^F-FDOPA PET and PET/CT have been investigated to address this problem. Several studies have directly compared the performance of contrast enhanced MRI with ^18^F-FDOPA PET or PET/CT in the identification of residual and recurrent gliomas. Jora et al. [23] reported ^18^F-FDOPA PET to have higher overall sensitivity and specificity over MRI, although their sample size was too limited to show any statistical significance. Karunanithi et al. [15] proved significantly higher specificity of ^18^F-FDOPA PET in diagnosing recurrence than contrast enhanced MRI overall (*p*-value = 0.002), and for HGGs (*p*-value = 0.006) and LGGs (*p*-value = 0.004) respectively. Besides the evidence presented above, our systematical analysis has also shown that ^18^F-FDOPA PET and PET/CT provide more reliable results in discriminating recurrent lesions from treatment related changes, with a pooled sensitivity and specificity of 0.92 and 0.76.

Another advantage of ^18^F-FDOPA PET and PET/CT is the utility in the detection of HGGs. Due to the differences between LGGs and HGGs in therapeutic regimen and prognosis evaluation, initial grading for gliomas through imaging approaches is of great significance. This meta-analysis proved the high overall value of ^18^F-FDOPA PET and PET/CT in evaluating tumor grade, with the AUC of 0.89. However, studies with respect to grading of recurrent gliomas demonstrated controversial results. In Fueger et al. [12], ^18^F-FDOPA uptake manifested no significant correlation with different tumor grades in recurrent gliomas, whereas Nioche et al. [32] reported a threshold of 1.8 to identify HGGs in pre-treated patients, with a sensitivity and specificity of 1.0 and 0.80 respectively. The discrepancy between limited studies concerned with grading recurrent gliomas made it unachievable for subgroup analysis. It is also worth noting that in Fueger et al. [12], only the data of newly-diagnosed group is available for meta-analysis. The exclusion of recurrent group might therefore introduce bias. In addition, since most patients included in our study are with newly-diagnosed gliomas (90%), our synthetic results are not representative enough for grading accuracy within the recurrent population, which still requires further investigation with larger sample size.

Evidence for comparisons of ^18^F-FDOPA with other PET tracers are available in several existing studies. As the most predominant PET tracer in oncological diagnostics so far, ^18^F-FDG shares the feature of being transported through integrate BBB. However, the high glucose metabolism within normal brain parenchyma may considerably hamper the differentiation between brain tumor and nonneoplastic tissue [38]. ^18^F-FDOPA has demonstrated better contrast than ^18^F-FDG in tumor versus normal tissues surrounding, and a higher sensitivity for detecting brain tumors than ^18^F-FDG [5, 10, 23]. The superiority in sensitivity of ^18^F-FDOPA can be further proved in comparison with previous meta-analysis of ^18^F-FDG (pooled sensitivity of 0.38, 95% CI: 0.27–0.50) [39]. The longest established amino acid tracer for brain tumor imaging is [^11^C]-methyl-L-methionine (^11^C-MET). In the only study with direct comparison between ^18^F-FDOPA PET and ^11^C-MET, ^18^F-FDOPA performed equally well as ^11^C-MET in the imaging of brain tumors [11]. However, ^11^C-MET is being replaced by tracers labelled with fluorine-18 (half-life of 109.8 min) due to its short half-life (20 min). The use of another amino acid tracer O-(2-[^18^F]-fluorethyl)-1-tyrosine (^18^F-FET) has rapidly grown in recent years. A previous meta-analysis of 5 studies including 180 patients reported a sensitivity and specificity of 0.82 and 0.76 of ^18^F-FET PET for the differential diagnosis of primary brain tumor [40]. In later comparison studies revealed that ^18^F-DOPA and ^18^F-FET shared similar uptake pattern in primary and recurrent HGGs and had similar diagnostic accuracy in recurrent HGGs [31, 41]. However, compared with ^18^F-FET, the elevated physiological ^18^F-FDOPA uptake in striatum has been a concern which may limits its use to detect brain tumor with striatum involvement [5, 42, 43]. Therefore, Kratochwil et al. recommended ^18^F-FET to examine patients with possible involvement of basal ganglia irrespective of tumor grade. Similarly, Morana et al. [44] reported that the diagnostic ability of ^18^F-FDOPA PET/CT in dorsal striatum was concordant with its overall accuracy, while not in the ventral striatum, which required fused MRI to improve its performance.

Several study limitations also need to be addressed. Although 19 studies were included in this meta-analysis, the sample size of each study tended to be small, and the number of true negative cases was particularly limited, which might possibly yield less replicable results, especially the pooled specificity. Limited sample size also led to data loss in stratification of subgroups when true negative case was less than one (specificity could not be calculated in this case). Besides, multiple specimens were obtained from one patient in three studies [24, 27, 30], which impeded our analysis on a per-patient basis. In addition, different imaging protocols and grading parameters from separate studies limited the quantitative analysis we performed for glioma grading. Finally, since most studies included in our analysis were published before the WHO 2016 classification system [45], the correlation of ^18^F-FDOPA uptake with glioma molecular characteristics cannot be analyzed.

## Conclusions

In conclusion, this meta-analysis provides evidence that ^18^F-FDOPA PET and PET/CT have high diagnostic accuracy for the detection of gliomas, especially the discrimination between tumor recurrence and radiation-induced changes. ^18^F-FDOPA PET and PET/CT also demonstrate good grading performance for the distinction between HGGs and LGGs. However, the grading parameters needs further investigation with standardized imaging protocols in prospective studies.

## Data Availability

All data generated or analyzed during this study are included in this published article.

## Abbreviation

^11^C-MET: [^11^C]-methyl-L-methionine
^18^F-FDG: 18-fluoro-deoxyglucose
^18^F-FDOPA: 3,4-dihydroxy-6-[^18^F] fluoro-L-phenylalanine
^18^F-FET: O-(2-[^18^F]-fluorethyl)-1-tyrosine
AUC: Area under the curve
BBB: Blood brain barrier
CI: Confidence intervals
CT: Computed tomography
DOR: Diagnostic odds ratio
FN: False negative
FP: False positive
HGGs: High-grade gliomas
LGGs: Low-grade gliomas
LR-: Negative likelihood ratio
LR +: Positive likelihood ratio
MRI: Magnetic resonance imaging
PET: Positron emission tomography
QUADAS-2: Quality Assessment of Diagnostic Accuracy Studies 2
SE: Standard Error
SROC: Summary receiver operating characteristic
TN: True negative
TP: True positive
WHO: World Health Organization
